# Who Reaches the NHL? A 20-Year Retrospective Analysis of Junior and Adult Ice Hockey Success in Relation to Biological Maturation in Male Swedish Players

**DOI:** 10.1007/s40279-023-01985-z

**Published:** 2024-01-09

**Authors:** Erik Niklasson, Oliver Lindholm, Marlene Rietz, John Lind, David Johnson, Tommy R. Lundberg

**Affiliations:** 1 Division of Clinical Physiology, Department of Laboratory Medicine, ANA Futura, Karolinska Institutet, Huddinge, 14152 Stockholm, Sweden; 2 Research Unit for Exercise Epidemiology, Department of Sports Science and Clinical Biomechanics, Center for Research in Childhood Health, University of Southern Denmark, Odense, Denmark; 3 Swedish Ice Hockey Association, Stockholm, Sweden; 4 Department for Health, University of Bath, Bath, England, UK; 5 Unit of Clinical Physiology, Karolinska University Hospital, Stockholm, Sweden

## Abstract

**Objectives:**

This study investigated the relationship between biological maturation and success in adolescence and adulthood in male Swedish ice hockey players.

**Methods:**

Anthropometric records of players in certified ice hockey high schools between 1998 and 2017 were retrieved (*n* = 4787). The database was complemented with records of Swedish junior national teams (U16, U18, U20) and National Hockey League (NHL) appearances. Biological maturation was recorded as a percentage of adult height (%AH), and selection probabilities were estimated using a generalised linear mixed effects model. Biological age was determined by comparing players with age-matched growth reference values. Categories of %AH, standard deviation *z*-scores and biological age offset describing early, on-time and late maturation were created.

**Results:**

A total of 217 players had played on the U16 national team (junior success), and 96 reached the NHL (adult success). The difference [95% confidence interval (CI)] in baseline %AH between players with junior versus adult success was − 0.75 (− 0.39, − 1.11). Looking at age-offset categories in junior success, 30% of players were early maturing and 19% of players were late maturing, showing a bias towards early maturation (*p* < 0.01). In contrast, more late-maturing players (40%) achieved adult success than early-maturing players (25%), and NHL players had significantly later maturation [%AH: − 0.48 (− 0.80, − 0.16)] than non-NHL players.

**Conclusion:**

This unique 20-year analysis shows that junior success in male ice hockey is positively related to early maturation, while adult success is inversely related to advanced maturation. Ice hockey organisations should implement maturation assessments to optimise the development of both late- and early-matured players.

**Supplementary Information:**

The online version contains supplementary material available at 10.1007/s40279-023-01985-z.

## Key Points

**Table:** 

Growth and maturation may influence team selection during adolescence.
We report, for the first time, that selection of the first junior national team in elite Swedish ice hockey is related to early maturation, while adult success, i.e. reaching the NHL, is inversely related to advanced maturation timing.
Ice hockey associations, clubs and coaches should consider implementing biological maturation measures to support player development and talent identification processes.

## Introduction

Ice hockey is a physically demanding sport that requires a range of physiological and technical skills, including strength, speed, aerobic and anaerobic capacity, and tactical skills [1, 2]. These demands are set by the high-intensity and intermittent nature of the sport, which involves repeated accelerations, decelerations and changes in direction, as well as physical contact over three 20-min periods [3]. Therefore, physical fitness is a critical factor in ice hockey success. Longitudinal studies show that players in the National Hockey League (NHL)—considered the best ice hockey league in the world and based in North America—have seen an increase in average height, weight, strength and lean body mass over the past few decades [4, 5].

In youth sports, biologically more mature adolescents are significantly larger (stature and mass) and have better athletic performance (strength, power and aerobic fitness), compared with their peers of the same age [6]. Therefore, it may be difficult to accurately compare and evaluate the performance of young players, because the physical advantages of advanced biological maturation are favoured in selection and promotion opportunities over long-term athletic potential [7, 8]. This can have several implications for younger and less mature players as well as for the overall development of youth sports.

Previous research has shown that in physically demanding sports, such as soccer and rugby, there is a selection bias towards early-maturing athletes that increases with the onset of puberty and accelerates further at the elite level [9]. To our knowledge, the effects of maturation on team selection in ice hockey have not yet been studied in depth. However, one study found that ice hockey players selected for a Canadian provincial men's final team (14–15 years old) were taller, heavier and more mature than unselected players and age-matched controls [10]. This suggests that team selectors may favour early-maturing male ice hockey players [10]. While selecting more mature youth players may lead to early competitive success, it may be detrimental to the players' long-term development. If clubs and associations overlook later-maturing players, they risk neglecting a significant portion of the available talent pool and reduce the number of high-potential players who can reach the elite levels [11, 12].

The aim of this study was twofold: first, to investigate the relationship between biological maturity and success at the junior level as manifested in selection for the U16 national team in Swedish ice hockey; second, to examine how biological maturity relates to success in adulthood, i.e. reaching the NHL. The context of Swedish ice hockey is particularly well suited for this retrospective analysis, as we have access to a unique 20-year collection of data from certified ice hockey schools, including both anthropometric and performance data from junior national teams and the NHL. We hypothesised that there would be a bias towards early-maturing players among players selected to the U16 national team (i.e. junior success) and that this bias would persist among players reaching the NHL (i.e. adult success), because the pool of late-maturing players likely is very small.

## Methods

### Player Database

Data were collected from high schools (*n* = 37) with an ice hockey educational programme certified by the Swedish Ice Hockey Association between 1998 and 2017. Ice hockey players can apply to ice hockey high school programmes nationwide during their final year of elementary school. It is part of the general education system and allows players to combine regular education with ice hockey practice several times a week during school hours. The programme starts the year players turn 16 and concludes the year they turn 19. High schools (3 years of education) were required to subject each player biannually to a testing protocol that included anthropometrics. The Swedish Ethical Review Authority approved the retrospective analysis of this study (access number: 2021–03464). Digital spreadsheets were collected from the association and merged during data processing. All data entries in the database were manually reviewed to ensure unique identification. If a player represented different clubs during data collection, the player’s unique career trajectory was double-checked using an open-access ice hockey database [13]. Unidentifiable data entries were removed as longitudinal data for these data points were not available, and there was no guarantee that the control group would include junior national teams or NHL players. Female players (*n* = 13) were excluded from this study. A total of 4787 unique players with 22,293 individual testing occasions were included in the database post-clearance.

### Records of Players from Swedish Junior National Teams and the NHL

A list of names, clubs, playing positions, dates of birth, and positions of players who had played with junior national teams for players below the age of 16 (Team 16), 18 (Team 18) and 20 years (Team 20) was added using a publicly available database [14–16]. Information on participation in junior national teams was added to the high-school database. For all junior national teams, the data were available for players born between 1982 and 1998. We identified 569, 589 and 532 names for Team 16, Team 18 and Team 20, respectively, of which 367, 350 and 331 names were identified in the database, respectively. A list of 283 names, birth dates, adult height and positions of all Swedish players who played at least one NHL game between 1998 and 1999 through the 2021–2022 NHL season was compiled from a publicly available database [17], and 299 Swedish players were retrieved from NHL’s official website [18]. A total of 112 unique NHL players were identified in the database, and the collected information was added to the database. Players who had played in the NHL were matched to the high school and national team databases.

### Maturity Estimation

Body height and weight were measured by the school coaches to the nearest centimetre and 0.1 kg, respectively. A variable describing maturation was computed by calculating the percentage of players’ adult height (%AH) by dividing the baseline height (first semester) by the players’ final height in the sixth semester. Adult height derived from the online database [17] was used for NHL players missing sixth-semester height assessment. %AH was used as the classification of biological maturation which correlates well with skeletal age and is considered one of the best available non-invasive methods for assessing somatic maturity [19]. Thus, %AH is similar to that of the Khamis–Roche method, which is based on age 18 as adult height [20]. To assess maturity timing based on %AH, elite players (NHL and junior national team players) were classified as early, on-time or late maturity, based on comparisons with age-matched Swedish reference data [21]. No birth dates were available for non-elite participants in the larger database. On-time maturation was classified as standard deviation (SD) thresholds (*z*-scores) between − 0.5 and 0.5, and early and late maturation corresponded to *z*-scores > 0.5 and <  − 0.5, respectively [9]. In the control population [21], this would theoretically result in three roughly equal-sized groups. Biological age was calculated for each individual to the nearest decimal place by comparing the percentage of adult height (%AH) with the corresponding values in the Swedish reference data [21]. Specifically, the players' biological age was determined by comparing their %AH with the corresponding chronological age in a reference population. The age thus determined in the reference population was designated as the player's biological age [22, 23]. Chronological age at the time of collection was calculated to the closest decimal for all players with birthdates. Age offset was calculated for the elite sample using the difference between biological age and chronological age, and discrete classifications were computed by dividing age offset into early, on-time and late maturation using thresholds set to ± 0.5 years. Owing to errors in data input, a few players presented %AH > 100% at baseline. Therefore, players with %AH between 100 and 102% were assigned 100% (*n* = 103), as negative differences in height from baseline to follow-up were likely due to measurement errors. All players with %AH > 102% (*n* = 20) were cross-checked and corrected using a publicly available database [13], and players with %AH > 102% post-correction were excluded from the sample due to unreliable baseline data (*N* = 6). Growth trajectories and adult height [13] for players with severely late maturation estimates were additionally examined manually to exclude incorrect classifications, and the adult height for three additional participants was corrected.

### Statistical Analysis

The mean ± SD for the sample characteristics was tabulated across NHL selection. Baseline differences (i.e. first-semester data at age 16) were compared between those who reached NHL and those who did not reach NHL using independent two-sample *t*-testing. Moreover, descriptive information on the differences between high school players who were lost to follow-up and players in the retrospective cohort was presented. An approximately normal distribution of the maturity markers was confirmed using density plots. Differences in adult height, age offset and *z*-scores of %AH between NHL players with recovered adult height and height based on the sixth semester were investigated using independent two-sample *t*-testing. Differences in continuous *z*-scores of %AH and age offset between elite cohorts (teams 16, 18 and 20, and NHL) were analysed using one-way analysis of variance (ANOVA). Additionally, %AH and age offset were compared between elite and non-elite players using a two-sample *t*-test again. In an exploratory analysis, junior and adult success probabilities were modelled by maturation and relevant confounders using a generalised linear mixed effects (GLME) model with binomial probability distribution function and logit link function. In likelihood ratio testing, superiority of the chosen model over a univariable model was confirmed. Goodness of fit of relevant models was assessed using Akaike Information Criterion (AIC). For Team 16, the GLME model consisted of Team 16 selection (‘yes’/‘no’) as a binary response variable, and %AH at baseline and year of data collection as fixed effects, in addition to high school region as a random effect. For adult success probability estimation, the GLME included the binary response variable NHL selection as well as the same fixed and random effects as the Team 16 GLME, although it was additionally adjusted for selection to teams 16, 18 and 20, as participation in junior national teams likely predisposes individuals to a higher probability of NHL selection. All statistical analysis was performed using R (Version 4.2.3), and the packages ggplot2 [24], dplyr [25], ggpubr [26], tidyverse [27], lmtest [28] and tidyr [29] were used for data preparation and visualisation, while the glmer function from the lme4 package [30] was employed for GLME modelling.

## Results

### Study Population

**Fig. 1:**
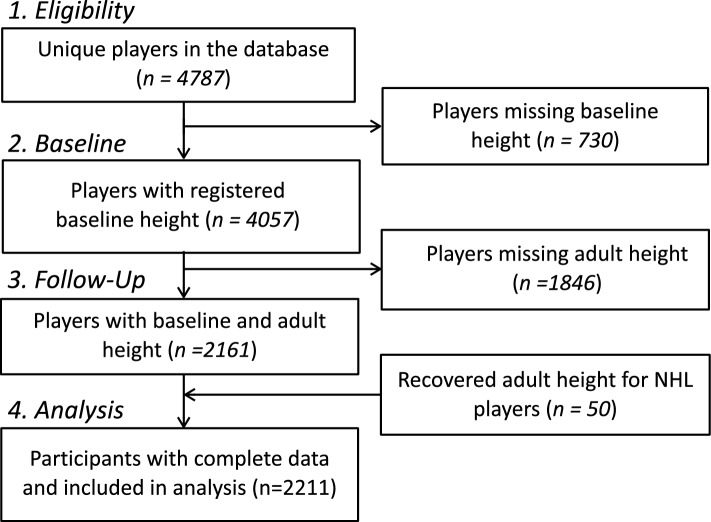
Flowchart of retrospective sample

The database included 4787 unique players of which 2211 participants had complete data to estimate maturation status. A detailed flow chart is shown in Fig. 1. For elite cohorts 217, 198, 200 and 96 players were identified in Team 16, Team 18, Team 20 and NHL, respectively. The mean biological age (± SD) at baseline was 16.5 (± 0.9) with the minimum and maximum corresponding to 13.6 and 18 years, respectively. Sample characteristics across participants with loss to follow-up and complete information are presented in Online Resource 1, Supplementary Table 1. Excluded junior players who did not have final adult height recorded presented similar baseline height and weight as the included players, suggesting an equal distribution of maturity. Players with loss to follow-up were significantly less likely to be selected to junior national teams and the NHL (Online Resource 1, Supplementary Table 1).

Baseline sample characteristics are presented in Table 1. Moreover, 56 NHL players reported follow-up height in combination with recovered adult height and 40 players presented recovered height only. There was no significant difference in age offset (*p* = 0.11) and *z*-scores of %AH (*p* = 0.13) between players with recovered or reported height during the final semester. Overall, NHL players were significantly taller and heavier at baseline than controls, whereas the body mass index (BMI) was similar (*p* = 0.18). Individuals reaching the NHL were more likely to be selected for all junior national teams (teams 16, 18 and 20; Table 1).

**Table 1:** Characteristics of retrospective sample across NHL success status

	No NHL (*n* = 2115)	NHL success (*n* = 96)	*p* value
*Anthropometrics*			
Height (m)	1.79 ± 0.06	1.83 ± 0.06	< 0.01
Weight (kg)	73.9 ± 8.0	77.4 ± 7.0	< 0.01
BMI (kg/m^2^)	23.0 ± 2.0	23.2 ± 1.7	0.18
*Career*			
Team 16 selection			< 0.01
Yes	169 (8%)	48 (50%)	
No	1946 (92%)	48 (50%)	
Team 18 selection			< 0.01
Yes	128 (6%)	70 (73%)	
No	1987 (94%)	26 (27%)	
Team 20 selection			< 0.01
Yes	112 (5%)	88 (92%)	
No	2003 (95%)	8 (8%)	

Anthropometric measurements (mean ± SD) at baseline and career success status (total *n* and % of sample) presented for adult success (NHL status). Differences were investigated using *t*-tests for continuous are variables and *X*^2^ tests for categorical variables. *p* values are presented for each characteristic

*NHL* National Hockey League, *BMI* body mass index

### Maturity Timing and Age Offset Across Elite Levels

**Fig. 2:**
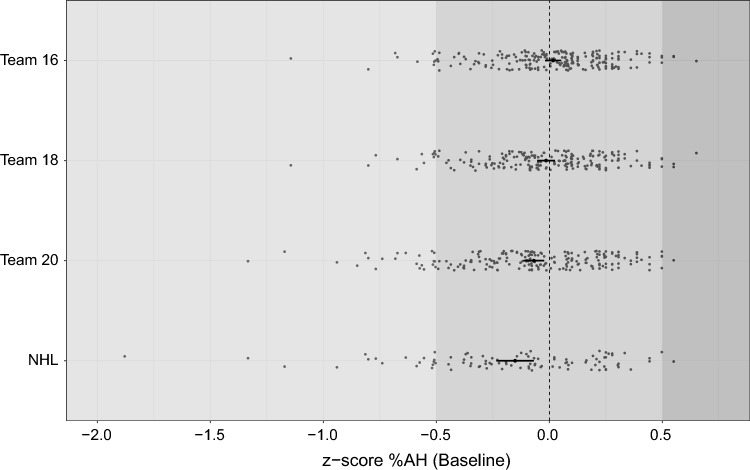
Distribution of *z*-score of %AH across subgroups. Dots are individuals, and bars represent the means for the respective groups. The dashed line represents the mean Swedish reference population. Dark-, medium- and light-grey backgrounds represent early, on-time and late maturation, respectively

%AH significantly differed across Team 16, Team 18, Team 20 and the NHL (ANOVA, *p* < 0.01). The estimated mean differences (95% CI) between individual teams are presented in Online Resource 1, Supplementary Table 2. Briefly, the mean %AH (95% CI) was significantly lower in the NHL group than in Team 16 and Team 18, and in Team 20 than in Team 16. Moreover, significantly earlier maturation (%AH) was determined for players that were selected for national Team 16 (*p* < 0.001) and Team 18 (*p* < 0.05) than for players that were not selected. The mean difference (95% CI) between non-selected and selected players was numerically larger for Team 16 [0.32 (0.18, 0.46)] than for Team 18 [0.15 (0.00, 0.31)]. There was no significant difference in the *z*-scores of %AH across Team 20 (*p* = 0.17). However, players reaching the NHL presented a significantly later maturation than those who did not reach NHL (mean difference of − 0.48 (− 0.80, − 0.16; *p* < 0.01)). The sample distribution and *z*-scores of %AH across the elite teams are shown in Fig. 2. Additionally, the mean *z*-scores for %AH were divided into categories describing early, on-time and late maturation (Online Resource 1, Supplementary Fig. 1).

**Fig. 3:**
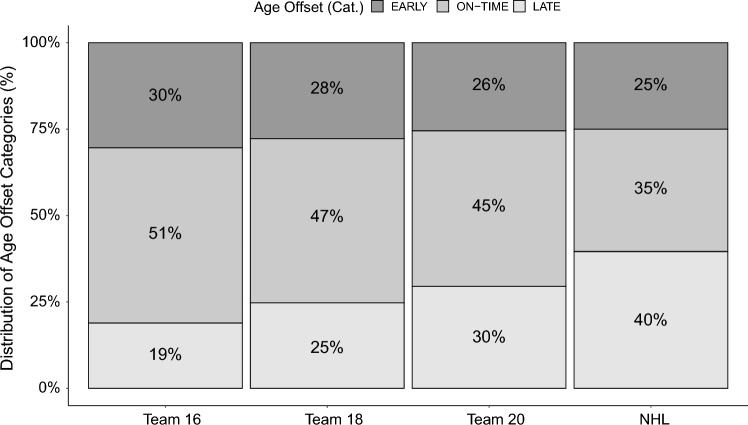
Distribution of the sample across age offset maturity categories and subgroups. The distribution of age offset maturity timing categories (late, on-time and early) across elite levels. Dark, medium and light grey represent early, on-time and late age offset, respectively

**Fig. 4:**
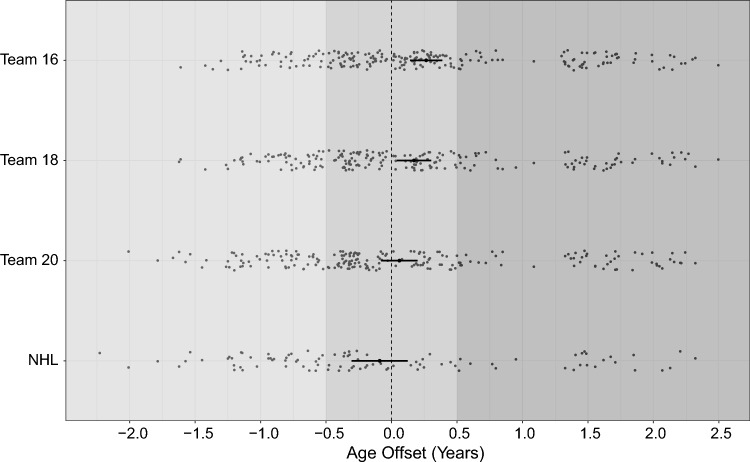
Age offset (years) (95% CI) across teams 16, 18 and 20, and NHL. Mean age offset in the respective group is highlighted including error bars representing the 95% confidence interval.The dashed line marks an age offset of 0.0 as defined using a Swedish reference dataset. Individual estimates are shown as a scatterplot. Dark-, medium- and light-grey backgrounds represent early, on-time and late maturation, respectively

Distributions of the samples across early-, on-time- and late-maturity age offset categories were visualised for all elite subgroups (Fig. 3). According to age offset, players selected for Team 16 were biologically older than the players not selected (*p* < 0.01; Fig. 4), while continuous age offset in other elite teams did not significantly differ from non-elite players (*p* > 0.05). The mean difference of age offset was − 0.25 (− 0.33, − 0.07) between selected and non-selected Team 16 players. Moreover, the mean age offset significantly differed between players selected for Team 16 and reaching NHL (ANOVA, *p* = 0.01), while other mean differences between elite subgroups were not significant (Online Resource 1, Supplementary Table 3).

### Probability of Junior and Adult Success

In addition to the more descriptive analyses described above, we carried out an explorative analysis to model junior and adult success probability based on biological maturation and relevant confounding variables using GLME models.

**Fig. 5:**
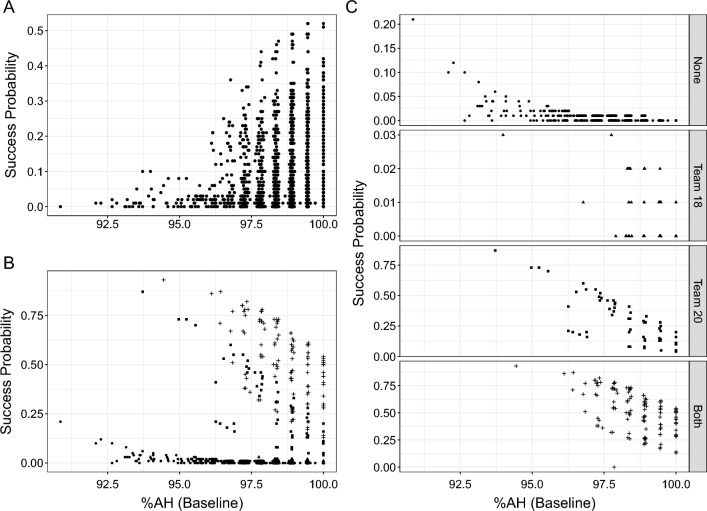
Estimates of probability of national Team 16 and adult success by maturity timing. **A** Probability estimates from a GLME model for Team 16 selection and *z*-scores of %AH at baseline adjusted for the year of data collection with high school as random effect. **B** Probability estimates from a GLME model for adult success (reaching the NHL) and *z*-scores of %AH at baseline adjusted for selection to teams 16, 18 and 20 and year of data collection, and high school region as random effect. Estimates are represented as: filled circle, did not play in Team 18 or Team 20; filled triangle, played in Team 18; filled square, played in Team 20; +, played in both teams. **C** Probability estimates for NHL status from **B** across participation in national teams 18 and 20

Overall, 9.8% (*n* = 217) of the cohort with maturity estimations were selected for Team 16. Initially, superiority of the chosen GLME model over a univariable model was confirmed using likelihood ratio testing (Online Resource 1, Supplementary Table 4). Results of the model for Team 16 selection and maturation (%AH) highlighted a significant positive association of maturity with the probability of being selected for Team 16 at a fixed effect estimate (95% CI) of 0.21 (0.04, 0.37). Estimated probabilities for each individual player are shown in Fig. 5A. The year of selection was not a significant confounder of the selection probability for Team 16, but increased goodness of fit of the model (AIC 1221.3).

Moreover, 96 players, corresponding to 4.3% of the included players, reached the NHL. Raw proportions of players selected to the NHL by selection to junior elite teams can be obtained in Online Resource 1, Supplementary Table 5. Additionally, results of a likelihood ratio test highlighting a significantly better fit for the chosen model compared to the univariable model can be accessed in Online Resource 1, Supplementary Table 6. The GLME model for NHL success and biological maturation status (%AH) suggested a significant inverse association between biological maturation and probability estimates at a fixed effect estimate (95% CI) of − 0.50 (− 0.72, − 0.28) at an AIC of 372.8. Estimated individual probabilities by maturation is shown in Fig. 5B. The fixed effects of Team 18 (*p* = 0.001) and Team 20 (*p* < 0.001) selection were significant confounders of the association between biological maturation and reaching the NHL. Trends were contrary to Team 16 selection probabilities, and adjustment for Team 18 and 20 participation removed the predictive value of Team 16 selection for adult success (NHL status) within the model (*p* = 0.90). Probabilities for reaching the NHL were similar in players selected for Team 18 and Team 20 or Team 20 only, and weaker in players that were selected for no national teams or Team 18 only (Fig. 5C).

## Discussion

This study was motivated by the observation that physically demanding team sports tend to favour more mature players at a young age. In the retrospective analysis, we found significant differences in %AH during the first semester between junior success (national Team 16 selection) and adult success (reaching the NHL). Moreover, players selected for national teams 16 and 18 were significantly more mature than those not selected. Surprisingly, it was also reported that Swedish players reaching the NHL presented significantly lower %AH at age 16 than those not reaching the NHL. Employing GLME models, we highlighted that early maturation was positively associated with the probability of selection to national Team 16, while it was inversely related to the probability of reaching the NHL.

Maturity bias reduced with age and was inversed for players reaching the NHL. Consequently, the conversion rate of late-maturing players to the NHL was exceptionally high, while the conversion rate of early-maturing players was significantly lower. This was further highlighted by the Team 16 selection, which was skewed towards early-maturing players. However, Team 16 selection had no statistically significant impact on the probability model for reaching the NHL. The higher conversion rate among late-maturing players is consistent with a small study in Serbian elite football [12]. There are several possible explanations for this observation. Late maturers who keep playing may be more talented and face increased physical challenge, as they play against more mature individuals. The high probability of adult success among late-maturing players who have managed to stay in the system is also partly consistent with the ‘underdog hypothesis’ [11, 31]. The underdog hypothesis states that, although there is a strong relative age effect (RAE) in the junior elite towards an early birth date, this is partly offset in adulthood [31]. However, RAE and maturity timing effect are different concepts [32], in short, the effect of relative age being merely a calendar effect that already occurs in childhood, whereas the effect of maturity timing accelerates at puberty and is associated with the timing of puberty rather than the date of birth. Nonetheless, the underdog hypothesis may certainly apply to biological maturation as well, because RAE only causes differences up to 12 months, while the difference in biological age span in our sample is 4.4 years. In addition, our conversion rates by maturity timing are supported by a previous RAE study on NHL draftees [33], but methodological differences are important to consider.

The sample characteristics support previous reports that elite players are taller than lower-level players [34], as was true for NHL players compared with the rest in the current sample. Therefore, they likely had very little, if any, physical disadvantage at age 16, despite being later maturing players on average. This could also explain why so many late-maturing players in those years survived and eventually reached the NHL. This has implications for talent identification in ice hockey, where it could be beneficial to differentiate between height and maturation. Collectively, our data suggest that ice hockey organisations and clubs should improve their player development pathway so that both early and late developers receive training and support appropriate to their maturity level.

The national teams differed significantly in terms of maturity timing. The findings show that teams 16 and 18 favour early-maturing players, Team 20 is rather bias-free, and late-maturing players were overrepresented in the NHL group. This longitudinal maturity bias shift from early to late with older age was found in all categorisations including age offset and *z*-scores, although differing slightly in timing. Physical advantages of being an early-matured individual likely skew the selection for younger junior elite teams, whereas true potential is better represented in NHL selection. While Team 20 was less maturity biased, there may be biases towards selecting players that have been in the younger national teams, while late maturers do not get similar spotlights. The probability models further support these findings, showing a bias towards early maturers for Team 16 selection, while the opposite applied for NHL status. It should be noted that the NHL draft has a different cut-off in terms of date, where those born late in the year enter the draft the following year, allowing more time for players who happen to be both late-maturing and relatively young. Also, scouts usually watch players for several years before the draft. So, if the late-maturing players are good enough to get into an ice hockey high school, it could result in the late developers getting a proper evaluation as well. This could suggest that if you pass the first selection for high school hockey centres, later maturation is not a disadvantage, but rather benefits skill development by training with more mature players.

### Relevance to Clinical Practice

To mitigate the impact of maturity, bio-banding is a strategy to improve player development for both late- and early-maturing players [35, 36]. To the best of our knowledge, bio-banding has not yet been systematically evaluated in ice hockey. However, it has shown great promise in presenting new challenges to youth soccer players [37]. Instead of chronological age, players are grouped according to their maturity levels. This means that late-maturing players play against chronologically younger but maturity-matched peers, whereas early maturers play against chronologically older but maturity-matched peers. Therefore, late-maturing players can demonstrate leadership skills, become more involved in the game and excel in their technical and tactical skills. On the other hand, early-maturing players cannot rely on their physique and are therefore more challenged to develop technical skills to adapt to a more team-oriented style of play [35, 36].

Another strategy for identifying and selecting talent is to number shirts according to maturity [38]. This has been shown to reduce the maturity bias when assessing player potential, as well as towards relatively older players [38]. In addition, some soccer associations have developed a ‘future team’ concept in which parallel national teams, including late-maturing players, are selected [39, 40]. This could be useful in ice hockey, especially for national Team 16, as our findings indicate that Team 16, in its current form, may not add value to talent identification for adult success.

An obvious effect of maturity bias is that it may lead to a lack of opportunities for younger and less mature players. This may reduce enjoyment and sense of achievement and could risk compromising the overall development of youth sports. One reason for this maturity bias may be a narrow focus on success, and the lack of emphasis on developing a diverse and inclusive environment where participation, development, and long-term engagement are emphasised [41]. Coaches, club administrators and other decision-makers must consider the potential impact of maturity bias. Regular growth and maturation assessments in ice hockey could help professionals create a more developmentally appropriate environment for young athletes. To put this into daily practice, clubs can conduct regular anthropometric testing to determine maturation status and implement some of the above suggestions such as bio-banding and accounting for maturation biases, but also create tailored training programmes based on growth and maturation status, which may also reduce the growth-related increased risk of injury noted during the growth spurt [42, 43].

### Methodological Considerations

This study is unique because the sheer size of the dataset and the longitudinal follow-up allowed for sophisticated analyses. As in every observational study, this retrospective analysis is potentially limited by bias owing to residual confounding [44]. Although selection bias may be present in the study sample, similar maturation timing between players lost to follow-up was confirmed before commencing the analysis. A strength of the current study was the use of an appropriate reference group [21]. Most studies in the field refer to the Berkeley Growth Study [45] conducted in 1959. However, secular trends have shown an earlier and more pronounced growth spurt, particularly in boys [46]. Since these changes have been shown when comparing reference groups from 2002 [47] and 2014 [46], older reference groups, such as in the Berkley growth study [45], are likely to be skewed and overestimate the maturity bias if applied to youth cohorts born several decades later. Studies employing categorical cut-offs are heavily dependent on that the reference group is appropriately matched to the cohort being studied. Therefore, it is important for researchers to consider the suitability of reference groups and to use continuous variables whenever possible.

We recognise the limitation that causal inferences between biological maturation and success are difficult to draw and that factors such as training environment, coaching, resources and infrastructure can influence athletic development. Furthermore, selection procedures may differ not only between different ice hockey associations, but especially between ice hockey and various other sports. Generalising our findings to other sports should therefore be done with caution. It should also be acknowledged that the applicability of the results to female sports and female ice hockey may differ due to differences in maturation, selection procedures and development pathways.

## Conclusion

We report that the selection of the first junior national team in Swedish elite male ice hockey is positively related to early maturation, while adult success, i.e. reaching the NHL, is inversely related to advanced maturation at baseline. Ice hockey associations, clubs, and coaches should consider implementing biological maturation measures to support player development and talent identification processes.

### Supplementary Information

Below is the link to the electronic supplementary material.

Supplementary file1 (PDF 92 KB)

## Declarations

### Data availability

Selected data are available upon reasonable request from the corresponding author. The data are not publicly available because combinations of variables could compromise the identities of individual participants.
