# Supplementary material for: Who Reaches the NHL? A 20-Year Retrospective Analysis of Junior and Adult Ice Hockey Success in Relation to Biological Maturation in Male Swedish Players
Source: Sports Med. 2024 Jan 9;54(5):1317–26. doi: 10.1007/s40279-023-01985-z (PMC11127826; doi:10.1007/s40279-023-01985-z)
Supplement: Supplementary file 1 — Supplementary file1 (PDF 92 KB) [file 40279_2023_1985_MOESM1_ESM.pdf]

Supplementary Online Material, Sports Medicine

Who reaches the NHL? A 20-year retrospective analysis of junior and adult ice hockey success in relation to biological maturation in male players

Erik Niklasson [1], Oliver Lindholm [1], Marlene Rietz, BSc [1,2], John Lind [3], David Johnson, PhD [4], Tommy R Lundberg, PhD [1,5]

AFFILIATIONS
[1] Department of Laboratory Medicine, Division of Clinical Physiology, Karolinska Institutet, Stockholm, Sweden
[2] Center for Research in Childhood Health, Research Unit for Exercise Epidemiology, Department of Sports Science and Clinical Biomechanics, University of Southern Denmark, Odense, Denmark
[3] Swedish Ice Hockey Association, Stockholm, Sweden
[4] Department for Health, University of Bath, Bath, England, UK
[5] Unit of Clinical Physiology, Karolinska University Hospital, Stockholm, Sweden

Corresponding author:
Tommy Lundberg, PhD
Tommy.lundberg@ki.se

Table of Contents

Supplementary Table 1 - Sample characteristics across participants with loss-to-follow-up and complete information

Supplementary Table 2 - Comparison of mean z-scores of %AH (95% CI) between elite teams estimated using a Tukey post-hoc test

Supplementary Figure 1 - Distribution of the elite sample across z-score of %AH maturity categories across subgroups

Supplementary Table 3 - Comparison of mean age offset (95% CI) between elite teams estimated using a Tukey post-hoc test

Supplementary Table 4 - Likelihood Ratio Test: Team 16 Selection

Supplementary Table 5 - Proportion of players (%) selected to the NHL by Selection to Team 16, Team 18, and Team 20

Supplementary Table 6 - Likelihood Ratio Test: NHL Selection

Supplementary Table 1 – Sample characteristics across participants with loss-to-follow-up (Excluded) and complete information (Included)

		**Included** (n=2211)	**Excluded** (n=1846)	p-value
Anthropometrics	Height (m)	1.79 ± 0.06	1.79 ± 0.06	*<0.01*
	Weight (kg)	74.1± 8.0	73.9 ± 8.1	*0.48*
	BMI (kg/m^2)	23.0 ± 2.0	23.2 ± 2.0	*0.04*
Career	Team 16 selection			*<0.01*
	yes	217 (9.8%)	99 (5.4%)	
	no	1994 (90.2%)	1747 (94.6%)	
	Team 18 selection			*<0.01*
	yes	198 (9.0%)	107 (5.8%)	
	no	2013 (91.0%)	1739 (94.2%)	
	Team 20 selection			*<0.01*
	yes	200 (9.0%)	90 (4.9%)	
	no	2011 (91.0%)	1756 (95.1%)	
	National Team			*<0.01*
	yes	341 (15.4%)	168 (9.1%)	
	no	1870 (84.6%)	1678 (90.9%)	
	NHL selection			*<0.01*
	yes	96 (4.3%)	8 (0.4%)	
	no	2115 (95.7%)	1838 (99.6%)	

Supplementary Table 2 – Comparison of mean %AH (95% CI) between elite teams estimated using a Tukey post-hoc test

Team A	***Team B***	***Mean Difference (95%CI)***	***p-value***
Team 16	NHL	0.75 (0.39, 1.11)	<0.001
Team 18	NHL	0.60 (0.23, 0.97)	<0.001
Team 20	NHL	0.35 (-0.02, 0.71)	0.07
Team 18	Team 16	-0.15 (-0.44, 0.14)	0.55
Team 20	Team 16	-0.40 (-0.69, -0.11)	<0.01
Team 20	Team 18	-0.25 (-0.55, 0.04)	0.13

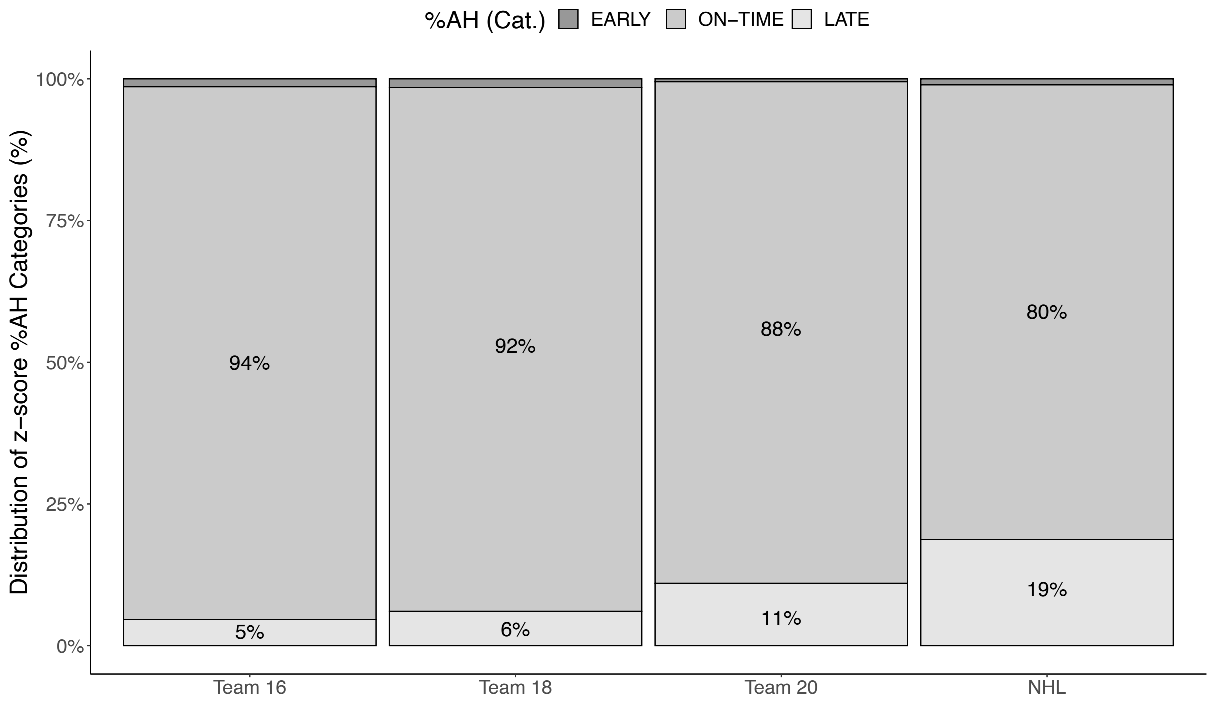

Supplementary Figure 1 – Distribution of the elite sample across z-score of %AH maturity categories across subgroups

Distribution of maturity timing categories (late, on time, and early) across elite levels. Dark, medium, and light grey represents early, on-time, and late z-score, respectively.

Supplementary Table 3 – Comparison of mean age offset (95% CI) between elite teams estimated using a Tukey post-hoc test

Team A	***Team B***	***Mean Difference (95%CI)***	***p-value***
Team 16	NHL	0.36 (0.05, 0.66)	*0.01*
Team 18	NHL	0.26 (-0.05, 0.57)	*0.13*
Team 20	NHL	0.15 (-0.16, 0.46)	*0.59*
Team 18	Team 16	-0.09 (-0.34, 0.15)	*0.76*
Team 20	Team 16	-0.20 (-0.45, 0.04)	*0.14*
Team 20	Team 18	-0.11 (-0.36, 0.14)	*0.66*

Supplementary Table 4 – Likelihood Ratio Test: Team 16 Selection

Chosen Model: team_16 ~ poah_term_1 + year + (1 | region)
Univariable Model: team_16 ~ poah_term_1

	Degrees of Freedom	***Log-Likelihood***	***Df***	***Chisq***	***Pr(>Chisq)***
Univariable	19	-591.65			
Multivariable	2	-701.65	-17	220	< 0.001

Supplementary Table 5 – Proportion of players (%) selected to the NHL by selection to Team 16, Team 18, and Team 20

	Selected	***Not Selected***
Team 16	*22.1%*	*2.4%*
Team 18	*35.4%*	*1,3%*
Team 20	*44.0%*	*0.4%*

Supplementary Table 6 – Likelihood Ratio Test: NHL Selection

Chosen Model: nhl_player ~ poah_term_1 + team_16 + team_18 + team_20 + year + (1 | region)
Univariable Model: nhl_player ~ poah_term_1

	Degrees of Freedom	***Log-Likelihood***	***Df***	***Chisq***	***Pr(>Chisq)***
Univariable	22	164.41			
Multivariable	2	-388.89	-20	448.95	< 0.001
